# ﻿Morphological and phylogenetic analyses reveal two new species of *Neohelicomyces* (Tubeufiales, Tubeufiaceae) from China

**DOI:** 10.3897/mycokeys.121.158721

**Published:** 2025-08-29

**Authors:** Xiao-Yan Ma, Dan-Dan Song, Jian Ma

**Affiliations:** 1 School of Food and Pharmaceutical Engineering, Guizhou Institute of Technology, Guiyang 550003, China School of Food and Pharmaceutical Engineering, Guizhou Institute of Technology Guiyang China; 2 Guizhou Key Laboratory of Agricultural Microbiology, Guizhou Academy of Agricultural Sciences, Guiyang 550009, China Guizhou Key Laboratory of Agricultural Microbiology, Guizhou Academy of Agricultural Sciences Guiyang China; 3 College of Brewing Engineering, Moutai Institute, Renhuai 564507, China College of Brewing Engineering, Moutai Institute Renhuai China; 4 Guizhou Industry Polytechnic College, Guiyang 550008, China Guizhou Industry Polytechnic College Guiyang China

**Keywords:** 2 new species, asexual morph, Dothideomycetes, hyphomycetes, taxonomy

## Abstract

*Neohelicomyces* is a genus of helicosporous hyphomycetes with the potential to produce bioactive secondary metabolites. During a survey of helicosporous fungi in Guizhou and Hainan provinces, southern China, four isolates were obtained from both freshwater and terrestrial habitats. Based on combined analyses of multigene phylogenetic data (ITS, LSU, *tef*1-α, and *rpb*2) and morphological characteristics, two novel species, *Neohelicomyces aquisubtropicus* and *N. wuzhishanensis*, are proposed. Detailed descriptions, illustrations, and phylogenetic analyses of the new taxa are presented. Additionally, a checklist of currently accepted *Neohelicomyces* species supported by molecular data is provided.

## ﻿Introduction

Luo et al. (2017) introduced the genus *Neohelicomyces* and designated *N. aquaticus* as the type species based on phylogenetic analysis of a combined dataset (*ITS*, *LSU*, and *tef*1-α) and morphological features. The asexual morph of *Neohelicomyces* is characterized by gregarious colonies that are white, grayish-brown, yellowish-green, or pinkish; macronematous, mononematous, erect, septate, pale brown, branched and/or unbranched conidiophores; mono- to polyblastic, denticulate, integrated, terminal or intercalary conidiogenous cells; and acropleurogenous or pleurogenous, aseptate or septate, guttulate, hyaline, helicoid conidia (Lu et al. 2018, 2022; Tibpromma et al. 2018; Crous et al. 2019a,b; Dong et al. 2020; Hsieh et al. 2021; Yang et al. 2023; Ma et al. 2024a, b; Peng et al. 2025).

Currently, *Neohelicomyces* comprises 28 species (Yang et al. 2023; Ma et al. 2024b; Peng et al. 2025; Sun et al. 2025). Among them, 12 species are found in freshwater habitats, 12 in terrestrial habitats, and four in both freshwater and terrestrial habitats (Table 1). *Neohelicomyces* species are distributed in China, the Czech Republic, Germany, Italy, Japan, the Netherlands, Thailand, and the USA (Hsieh et al. 2021; Yang et al. 2023; Ma et al. 2024b; Peng et al. 2025). They occur as saprobes on bamboo culms, *Deschampsia cespitosa*, *Fraxinus excelsior*, *Melaleuca styphelioides*, *Miscanthus floridulus*, *Pandanus* sp., *Quercus robur*, and decaying wood in both freshwater and terrestrial habitats (Linder 1929; Goos 1985, 1986, 1989; Tsui et al. 2006; Zhao et al. 2007; Ruibal et al. 2009; Luo et al. 2017; Lu et al. 2018, 2022; Tibpromma et al. 2018; Crous et al. 2019a, b; Dong et al. 2020; Hsieh et al. 2021; Yang et al. 2023; Ma et al. 2024b; Peng et al. 2025; Sun et al. 2025).

**Table 1.** A checklist of accepted *Neohelicomyces* species with molecular data.

No.	Species	Distribution	Habitat	Reference
1	*Neohelicomyces acropleurogenus*	China	Terrestrial	Ma et al. (2024b)
2	*Neohelicomyces aquaticus*	China	Freshwater	Luo et al. (2017)
**3**	***Neohelicomyces aquisubtropicus***	**China**	**Terrestrial**	**This study**
4	*Neohelicomyces aseptatus*	China	Terrestrial	Ma et al. (2024b)
5	*Neohelicomyces dehongensis*	China	Freshwater	Dong et al. (2020)
6	*Neohelicomyces denticulatus*	China	Freshwater	Yang et al. (2023)
7	*Neohelicomyces deschampsiae*	Germany	Terrestrial	Crous et al. (2019a)
8	*Neohelicomyces edgeworthiae*	China	Terrestrial	Ma et al. (2024b)
9	*Neohelicomyces guizhouensis*	China	Freshwater	Ma et al. (2024a)
10	*Neohelicomyces guttulatus*	China	Freshwater/Terrestrial	Ma et al. (2024b)
11	*Neohelicomyces grandisporus*	China	Freshwater	Luo et al. (2017)
12	*Neohelicomyces hainanensis*	China	Terrestrial	Lu et al. (2022)
13	*Neohelicomyces helicosporus*	China	Terrestrial	Ma et al. (2024a)
14	*Neohelicomyces hyalosporus*	China	Freshwater	Lu et al. (2018)
15	*Neohelicomyces hydei*	China	Freshwater	Ma et al. (2024a)
16	*Neohelicomyces lignicola*	China	Freshwater	Ma et al. (2024b)
17	*Neohelicomyces longisetosus*	China	Freshwater	Hsieh et al. (2021)
18	*Neohelicomyces macrosporus*	China	Freshwater	Ma et al. (2024b)
19	*Neohelicomyces maolanensis*	China	Terrestrial	Peng et al. (2025)
20	*Neohelicomyces melaleucae*	China, USA	Freshwater/Terrestrial	Crous et al. (2019b)
21	*Neohelicomyces pallidus*	China, Czech Republic, Italy, Japan, Netherlands, USA	Freshwater/Terrestrial	Linder (1929); Goos (1989); Tsui et al. (2001); Zhao et al. (2007); Lu et al. (2018); Ma et al. (2024b)
22	*Neohelicomyces pandanicola*	China	Terrestrial	Tibpromma et al. (2018)
23	*Neohelicomyces qixingyansis*	China	Terrestrial	Ma et al. (2024b)
	*Neohelicomyces sexualis*	China	Terrestrial	Sun et al. (2025)
24	*Neohelicomyces submersus*	China	Freshwater	Luo et al. (2017)
25	*Neohelicomyces subtropicus*	China	Terrestrial	Peng et al. (2025)
26	*Neohelicomyces thailandicus*	China, Thailand	Freshwater/Terrestrial	Dong et al. (2020); Ma et al. (2024b)
**27**	***Neohelicomyces wuzhishanensis***	**China**	**Freshwater**	**This study**
28	*Neohelicomyces xiayadongus*	China	Terrestrial	Ma et al. (2024b)
29	*Neohelicomyces yunnanensis*	China	Freshwater	Ma et al. (2024b)

Note: The newly isolated species in this study are highlighted in bold.

*Neohelicomyces* has the potential to produce secondary metabolites (Zheng et al. 2023). For example, two alkaloid compounds isolated from *Neohelicomyces hyalosporus* exhibited moderate cytotoxic effects on human cancer cells (Zheng et al. 2023).

In this study, four helicosporous hyphomycete isolates, representing two distinct taxa, were obtained from both freshwater and terrestrial habitats in Guizhou and Hainan provinces, China. Based on morphological characteristics, illustrations, and multigene phylogenetic analyses, two novel species are introduced: *Neohelicomyces aquisubtropicus* and *N. wuzhishanensis*.

## ﻿Materials and methods

### ﻿Sample collection, examination, and isolation

Decaying wood samples were collected from freshwater and terrestrial habitats in Guizhou and Hainan provinces, China, from August 2021 to April 2022. After the collection information was recorded (Rathnayaka et al. 2024), the fungal specimens were taken to the mycology laboratory at the Guizhou Institute of Technology for examination. Fresh specimens from freshwater habitats were cultured at room temperature, with moisture maintained for 1–2 weeks. Morphological characteristics were observed using a stereomicroscope (SMZ-168, Nikon, Japan) and photographed using an ECLIPSE Ni compound microscope (Nikon, Tokyo, Japan) equipped with a Canon 90D digital camera.

Single-spore isolations were conducted following the procedure outlined in Senanayake et al. (2020). Subsequently, the germinating spores were aseptically transferred to fresh potato dextrose agar (PDA). Dried specimens were deposited in the Herbarium of Kunming Institute of Botany, Chinese Academy of Sciences (Herb. HKAS), Kunming, China, and the Herbarium of Guizhou Academy of Agriculture Sciences (Herb. GZAAS), Guiyang, China. Pure cultures were deposited at the Guizhou Culture Collection (**GZCC**), Guiyang, China. The MycoBank numbers were obtained as described at https://www.mycobank.org/.

### ﻿DNA extraction, PCR amplification, and sequencing

Fresh fungal mycelia grown on PDA media for 39–45 days were scraped with a sterilized toothpick and transferred to a 1.5 ml microcentrifuge tube for genomic DNA extraction. Genomic DNA was extracted using the Biospin Fungus Genomic DNA Extraction Kit (BioFlux, China), following the manufacturer’s protocol. Primer pairs ITS5/ITS4 (White et al. 1990), LR0R/LR5 (Vilgalys and Hester 1990), EF1-983F/EF1-2218R (Rehner and Buckley 2005), and fRPB2-5F/fRPB2-7cR (Liu et al. 1999) were used to amplify ITS, LSU, *tef*1-α, and *rpb*2 sequence fragments, respectively. The PCR amplification reactions were carried out in a 25 µL reaction volume, including 1 µL of DNA, 1 µL each of the forward and reverse primers, and 22 µL of 1.1× T3 Super PCR Mix (Qingke Biotech, Chongqing, China). The polymerase chain reaction (PCR) followed the protocol reported by Ma et al. (2024a). The PCR products were detected by 1% agarose gel electrophoresis, and sequencing was performed at Beijing Tsingke Biotechnology Co., Ltd.

### ﻿Phylogenetic analyses

The forward and reverse sequence data of the new taxa were checked and assembled using BioEdit v. 7.0.5.3 (Hall 1999) and SeqMan v. 7.0.0 (DNASTAR, Madison, WI, USA; Swindell and Plasterer 1997), respectively. The sequences used in this study were downloaded from GenBank (Table 2; https://www.ncbi.nlm.nih.gov/). The single-gene datasets (ITS, LSU, *tef*1-α, and *rpb*2) were aligned using MAFFT v. 7.473 (https://mafft.cbrc.jp/alignment/server/, Katoh et al. 2019) and trimmed using trimAl v.1.2rev59 software (Capella-Gutiérrez et al. 2009). The aligned datasets (LSU–ITS–*tef*1-α–*rpb*2) were concatenated using SequenceMatrix-Windows v. 1.7.8 software (Vaidya et al. 2011). The maximum likelihood (ML) tree was constructed using the IQ-TREE webserver (http://iqtree.cibiv.univie.ac.at/, Nguyen et al. 2015).

**Table 2.** Taxa used in this study, along with their corresponding GenBank accession numbers.

Taxon	Strain	GenBank Accessions			
		ITS	LSU	*tef*1-α	*rpb*2
*Helicotubeufia hydei*	MFLUCC 17-1980^T^	MH290021	MH290026	MH290031	MH290036
*Helicotubeufia jonesii*	MFLUCC 17-0043^T^	MH290020	MH290025	MH290030	MH290035
*Muripulchra aquatica*	MFLUCC 15-0249^T^	KY320532	KY320549	-	-
*Neohelicomyces acropleurogenus*	CGMCC 3.25549^T^	PP626594	PP639450	PP596351	PP596478
***Neohelicomyces aquisubtropicus***	**GZCC 23-0080^T^**	**PQ098499**	**PQ098537**	**PV768327**	**PV768336**
***Neohelicomyces aquisubtropicus***	**GZCC 24-0163**	**PV730410**	**PV730414**	**PV768328**	**PV768337**
*Neohelicomyces aquaticus*	MFLUCC 16-0993^T^	KY320528	KY320545	KY320561	MH551066
*Neohelicomyces aseptatus*	CGMCC 3.25564^T^	PP626595	PP639451	PP596352	PP596479
*Neohelicomyces dehongensis*	MFLUCC 18-1029^T^	NR_171880	MN913709	MT954393	-
*Neohelicomyces denticulatus*	GZCC 19-0444^T^	OP377832	MW133855	-	-
*Neohelicomyces deschampsiae*	CPC 33686^T^	MK442602	MK442538	-	-
*Neohelicomyces edgeworthiae*	CGMCC 3.25565^T^	PP626597	PP639453	PP596354	PP596481
*Neohelicomyces grandisporus*	KUMCC 15-0470^T^	KX454173	KX454174	-	MH551067
*Neohelicomyces guizhouensis*	GZCC 23-0725^T^	PP512969	PP512973	PP526727	PP526733
*Neohelicomyces guttulatus*	CGMCC 3.25550^T^	PP626598	PP639454	PP596355	-
*Neohelicomyces hainanensis*	GZCC 22-2009^T^	OP508734	OP508774	OP698085	OP698074
*Neohelicomyces helicosporus*	GZCC 23-0633^T^	PP512971	PP512975	PP526729	PP526735
*Neohelicomyces hyalosporus*	GZCC 16-0086^T^	MH558745	MH558870	MH550936	MH551064
*Neohelicomyces hydei*	GZCC 23-0727^T^	-	PP512977	PP526731	PP526737
*Neohelicomyces lignicola*	CGMCC 3.25551^T^	PP626600	PP639456	PP596357	PP596483
*Neohelicomyces longisetosus*	NCYU-106H1-1-1^T^	MT939303	-	-	-
*Neohelicomyces macrosporus*	CGMCC 3.25552^T^	PP626601	PP639457	PP596358	PP596484
*Neohelicomyces maolanensis*	GZCC 23-0079^T^	-	PQ098529	PQ490683	PQ490677
*Neohelicomyces melaleucae*	CPC 38042^T^	MN562154	MN567661	MN556835	-
*Neohelicomyces pallidus*	CBS 271.52	AY916461	AY856887	-	-
*Neohelicomyces pallidus*	CBS 962.69	AY916460	AY856886	-	-
*Neohelicomyces pandanicola*	KUMCC 16-0143^T^	MH275073	MH260307	MH412779	-
*Neohelicomyces qixingyaensis*	CGMCC 3.25569^T^	PP626602	PP639458	PP596359	PP596485
*Neohelicomyces submersus*	MFLUCC 16-1106^T^	KY320530	KY320547	-	MH551068
*Neohelicomyces subtropicus*	GZCC 23-0076^T^	PQ098492	PQ098530	PQ490685	PQ490679
*Neohelicomyces thailandicus*	MFLUCC 11-0005^T^	NR_171882	MN913696	-	-
***Neohelicomyces wuzhishanensis***	**GZCC 23-0410^T^**	**PQ098494**	**PQ098532**	**PV768325**	**PV768334**
***Neohelicomyces wuzhishanensis***	**GZCC 24-0164**	**PV730409**	**PV730413**	**PV768326**	**PV768335**
*Neohelicomyces xiayadongensis*	CGMCC 3.25568^T^	PP626604	PP639460	PP596361	PP596487
*Neohelicomyces yunnanensis*	GZCC 23-0735^T^	PP664109	PP664113	-	-
*Tubeufia guttulata*	GZCC 23-0404^T^	OR030841	OR030834	OR046678	OR046684
*Tubeufia hainanensis*	GZCC 22-2015^T^	OR030842	OR030835	OR046679	OR046685
*Tubeufia javanica*	MFLUCC 12-0545^T^	KJ880034	KJ880036	KJ880037	-
*Tubeufia krabiensis*	MFLUCC 16-0228^T^	MH558792	MH558917	MH550985	MH551118
*Tubeufia latispora*	MFLUCC 16-0027^T^	KY092417	KY092412	KY117033	MH551119
*Tubeufia laxispora*	MFLUCC 16-0232^T^	KY092413	KY092408	KY117029	MF535287
*Tubeufia mackenziei*	MFLUCC 16-0222^T^	KY092415	KY092410	KY117031	MF535288
*Tubeufia muriformis*	GZCC 22-2039^T^	OR030843	OR030836	OR046680	OR046686
*Tubeufia nigroseptum*	CGMCC 3.20430^T^	MZ092716	MZ853187	OM022002	OM022001
*Tubeufia pandanicola*	MFLUCC 16-0321^T^	MH275091	MH260325	-	-
Tubeufiaceae sp.	ATCC 42524	AY916458	AY856911	-	-

Note: “^T^” indicates ex-type strains. Newly generated sequences are in bold. “-” indicates the unavailable data in GenBank.

Bayesian analyses were carried out using MrBayes v. 3.2.7a, and the best nucleotide substitution model for each data partition was selected using MrModeltest v. 2.3 under the Akaike Information Criterion (AIC) (Nylander et al. 2008). The aligned FASTA file was converted to a NEXUS format for Bayesian analysis using AliView v. 1.27 (Larsson 2014).

Phylogenetic trees were visualized using FigTree v. 1.4.4 and subsequently edited in Adobe Illustrator CC 2019 (v. 23.1.0; Adobe Systems, USA). Photo plates and scale bars were prepared using Adobe Photoshop CC 2019 (Adobe Systems, USA) and the Tarosoft Image Framework program, respectively.

### ﻿Phylogenetic analysis results

**Figure 1.**
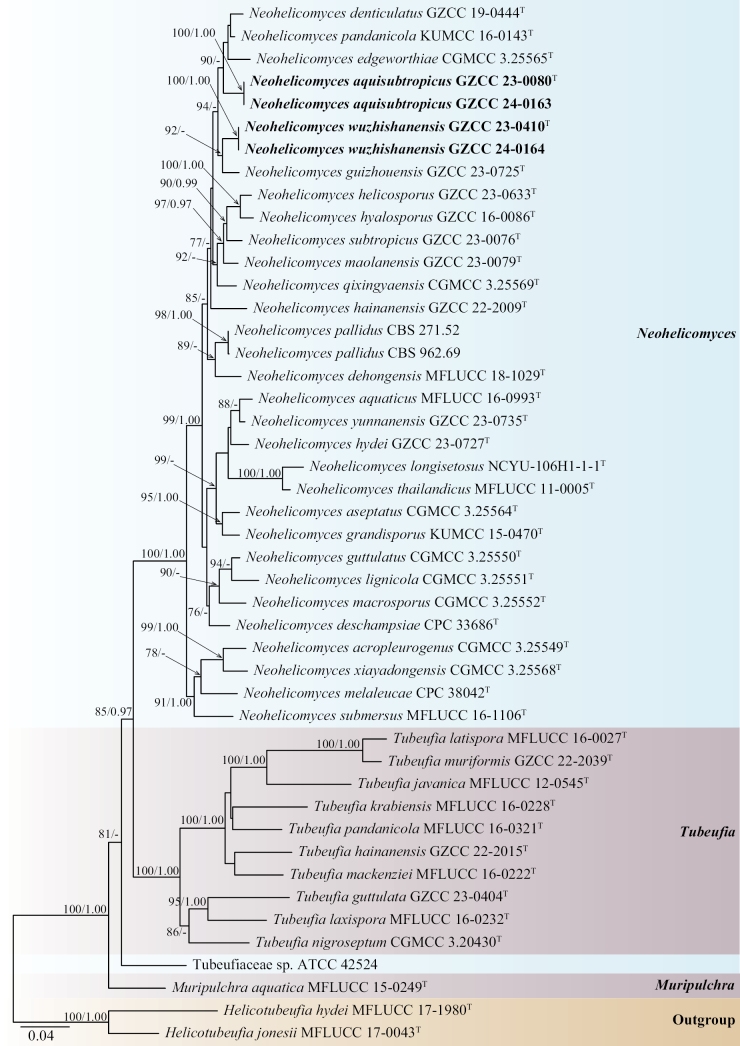
Phylogenetic tree generated from ML analysis based on the combined ITS, LSU, *tef*1-α, and *rpb*2 sequence data. Bootstrap support values for ML (≥ 75%) and BI (≥ 0.95) are indicated near their respective nodes. The tree is rooted with *Helicotubeufia hydei* (MFLUCC 17-1980) and *H. jonesii* (MFLUCC 17-0043). Ex-type strains are denoted with “T,” and newly obtained strains are in bold black fonts.

The phylogenetic placements of the four new strains were determined by multilocus phylogenetic analysis. The concatenated sequence matrix comprised 3,410 characters (ITS: 1–573, LSU: 574–1,430, *tef*1-α: 1,431–2,341, and *rpb*2: 2,342–3,410) across 46 taxa. Both ML and BI analyses produced congruent topologies. Fig. 1 presents the best-scoring ML tree, which had a final log-likelihood value of –19,563.192. Species delimitation and the introduction of new taxa were conducted following the taxonomic framework proposed by Chethana et al. (2021).

Based on the multigene phylogenetic tree (Fig. 1), our collections represent two distinct species of *Neohelicomyces* within the family Tubeufiaceae. Isolates GZCC 23-0080 and GZCC 24-0163 formed a sister clade to the clade comprising *Neohelicomyces denticulatus* (GZCC 19-0444), *N. edgeworthiae* (CGMCC 3.25565), and *N. pandanicola* (KUMCC 16-0143). Isolates GZCC 23-0410 and GZCC 24-0164 formed a clade that is sister to *Neohelicomyces guizhouensis* (GZCC 23-0725) with 92% ML bootstrap support.

## ﻿Taxonomy

### ﻿ Neohelicomyces aquisubtropicus

Taxon classificationFungiTubeufialesTubeufiaceae

X.Y. Ma, Y.Z. Lu & J. Ma sp. nov.

6CA725B7-D38A-5694-9E7F-411C2D3CC073

904050

Fig. 2

#### Etymology.

“aqui-’’ refers to the aquatic habitat of this fungus, and ‘‘-subtropicus’’ means the climate type where the fungus was collected.

**Figure 2.**
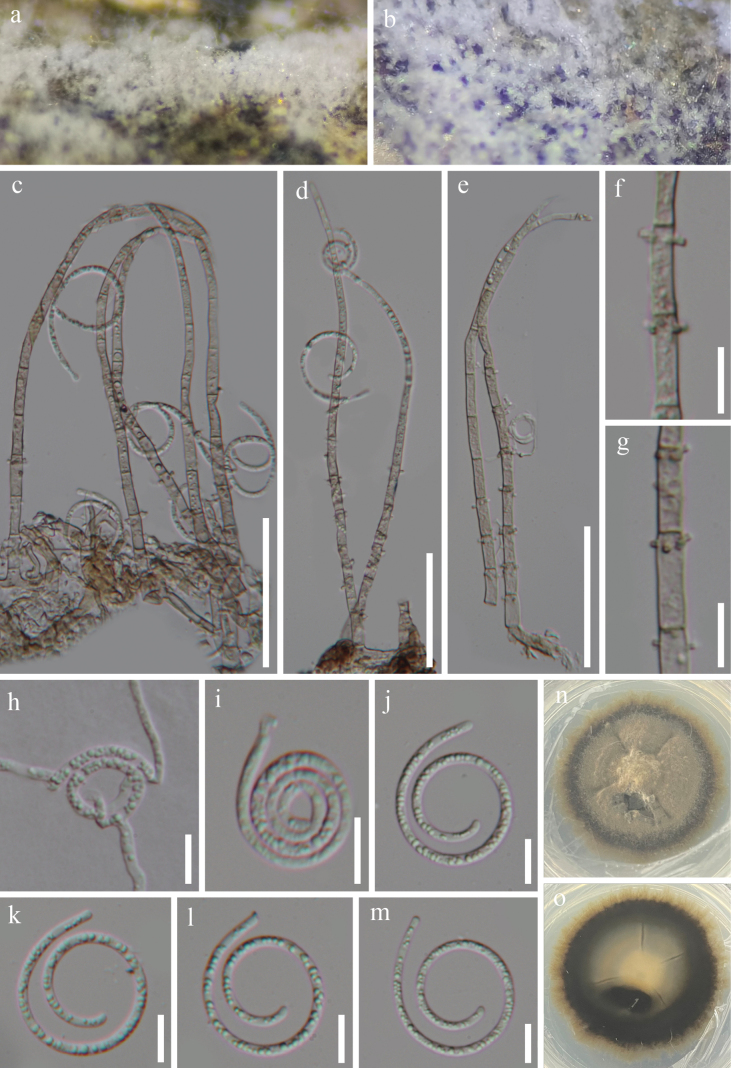
*Neohelicomyces aquisubtropicus* (HKAS 128947, holotype). **a, b.** Colonies on the host surface; **c–e.** Conidiophores, conidiogenous cells, and conidia; **f, g.** Conidiogenous cells; **h.** Germinated conidium; **i–m.** Conidia; **n, o.** Colonies on PDA from above and below after 45 days of incubation at room temperature. Scale bars: 20 μm (**c–e**); 10 μm (**f–m**).

#### Holotype.

HKAS 128947

#### Description.

***Saprobic*** on decaying wood in a terrestrial habitat. ***Sexual morph*** Undetermined. ***Asexual morph*** Hyphomycetous, helicosporous. ***Colonies*** on natural substrate superficial, white, effuse, gregarious, with massive glistening conidia. ***Mycelium*** partly superficial, composed of hyaline to pale brown, branched, septate, guttulate, smooth hyphae. ***Conidiophores*** 144–193.5 × 3.5–6.5 μm (x–¯ = 167.5 × 5 μm, n = 25), macronematous, mononematous, erect, cylindrical, straight or slightly flexuous, typically curved at the apex, unbranched, septate, subhyaline to pale brown, thick-walled. ***Conidiogenous cells*** 11.5–15 × 3.5–5 μm (x–¯ = 13.5 × 4 μm, n = 30), holoblastic, monoblastic, or polyblastic, integrated, intercalary, cylindrical, with denticles, subhyaline to pale brown, smooth-walled. ***Conidia*** solitary, pleurogenous, helicoid, tapering towards the rounded ends, developing on tooth-like protrusions, 14.5–17 μm diam., and conidial filament 2–4 μm wide (x–¯ = 15.5 × 3 μm, n = 25), 82.5–126.5 μm long (x–¯ = 105.5 μm, n = 30), tightly coiled up to 3^1^/_2_ times, becoming loosely coiled in water, aseptate, guttulate, hyaline, smooth-walled.

#### Culture characteristics.

Conidia germinate on PDA within 10 hours, producing germ tubes from the conidial body. Colonies on PDA are circular with a raised surface and entire margin, reaching 5 cm in diameter after 45 days at room temperature (approximately 25 °C), and are pale brown to dark brown on both the surface and reverse sides.

#### Material examined.

China • Guizhou Province, Qiannan Buyi and Miao Autonomous Prefecture, Libo County, on decaying wood in a terrestrial habitat, 10 April 2022, Jian Ma, MN6 (HKAS 128947, holotype), ex-type living cultures GZCC 23-0080; • *Ibid*., MN6.1 (GZAAS 24-0077, paratype), living culture GZCC 24-0163.

#### Notes.

Based on phylogenetic analyses, our isolates (GZCC 23-0080 and GZCC 24-0163) clustered with *Neohelicomyces denticulatus* (GZCC 19-0444), *N. edgeworthiae* (CGMCC 3.25565), and *N. pandanicola* (KUMCC 16-0143) (Fig. 1). *Neohelicomyces aquisubtropicus* (HKAS 128947) differs from *N. edgeworthiae* (HKAS 128877) in having smaller conidia (14.5–17 μm diam. and 82.5–126.5 μm long *vs.* 21.5–34 μm diam. and 121–177 μm long) (Ma et al. 2024b). Additionally, *N. denticulatus* (GZAAS 20-0339) and *N. pandanicola* (HKAS 96202) can be distinguished from *N. aquisubtropicus* (HKAS 128947) by their wider conidial diameters (16–22 μm and 28–44 μm *vs.* 14.5–17 μm) (Tibpromma et al. 2018; Yang et al. 2023). Moreover, base pair comparisons between *N. aquisubtropicus* (GZCC 23-0080) and related species reveal the following differences. Compared to *N. denticulatus* (GZCC 19-0444), there are 23/487 bp differences in ITS (4.7%, with 13 gaps). Compared to *N. edgeworthiae* (CGMCC 3.25565), there are 26/521 bp differences in ITS (5.0%, with 14 gaps), 21/929 bp differences in *tef*1-α (2.3%, with 7 gaps), and 27/811 bp differences in *rpb*2 (3.3%, with 10 gaps). In comparison with *N. pandanicola* (KUMCC 16-0143), there are 27/509 bp differences in ITS (5.3%, with 13 gaps) and 15/827 bp differences in *tef*1-α (1.8%, with 7 gaps). Therefore, we introduce *N. aquisubtropicus* as a new species based on morphology and multigene phylogenetic analysis.

### ﻿ Neohelicomyces wuzhishanensis

Taxon classificationFungiTubeufialesTubeufiaceae

X.Y. Ma, Y.Z. Lu & J. Ma sp. nov.

80BE7AFF-2F46-59B0-99AC-881ADA99853E

904051

Fig. 3

#### Etymology.

“*wuzhishanensis*” refers to the type location “Wuzhishan National Nature Reserve.”

**Figure 3.**
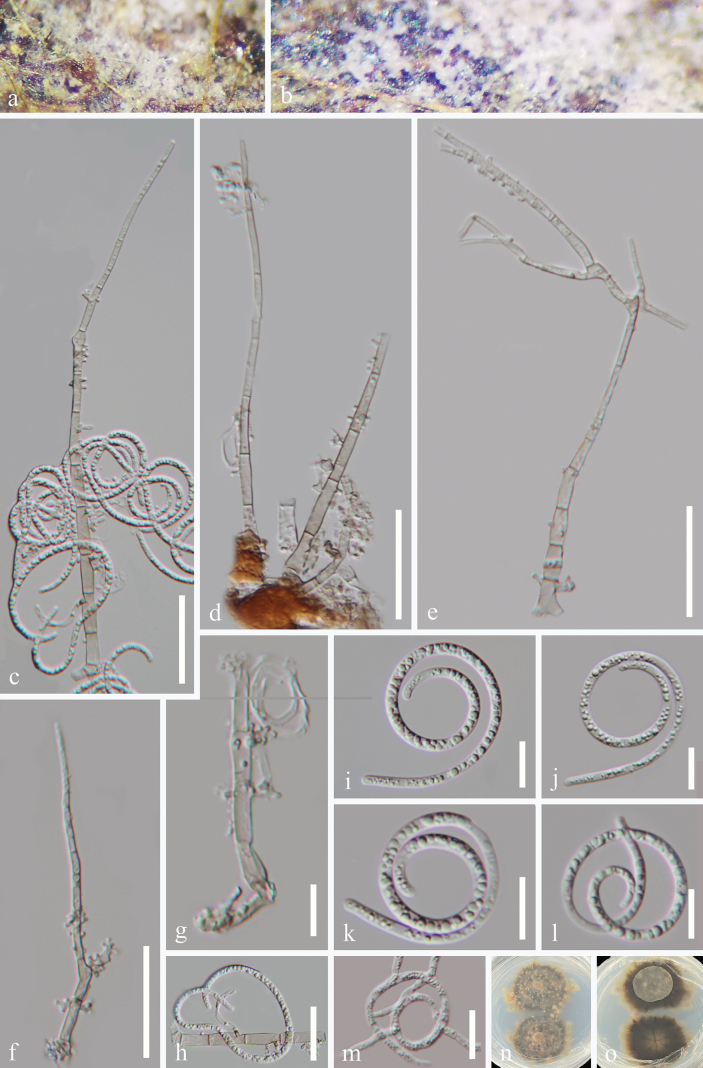
*Neohelicomyces wuzhishanensis* (HKAS 128942, holotype). **a, b.** Colonies on the host surface; **c–h.** Conidiophores, conidiogenous cells, and conidia; **i–l.** Conidia; **m.** A germinated conidium; **n, o.** Colonies on PDA from above and below after 39 days of incubation at room temperature. Scale bars: 30 μm (**c–f**); 20 μm (**h, m**); 10 μm (**g, i–l**).

#### Holotype.

HKAS 128942

#### Description.

***Saprobic*** on submerged decaying wood in a freshwater habitat. ***Sexual morph*** Undetermined. ***Asexual morph*** Hyphomycetous, helicosporous. ***Colonies*** on natural substrate superficial, effuse, gregarious, white. ***Mycelium*** partly immersed, composed of hyaline to pale brown, branched, septate, guttulate, smooth hyphae. ***Conidiophores*** 92–190 × 3.5–5 μm (x–¯ = 140 × 4.5 μm, n = 25), macronematous, mononematous, erect, cylindrical, widest at the base, tapering towards narrow apex, straight or slightly flexuous, occasionally branched, septate, subhyaline to pale brown, thick-walled. ***Conidiogenous cells*** 9.5–16.5 × 2.5–5 μm (x–¯ = 14 × 4 μm, n = 20), holoblastic, monoblastic, or polyblastic, integrated, intercalary or terminal, cylindrical, with tiny tooth-like or bladder-like protrusions, subhyaline to pale brown, smooth-walled. ***Conidia*** solitary, acropleurogenous, helicoid, tapering towards the rounded ends, developing on tooth-like protrusions, 23–26 μm diam., and conidial filament 2.3–3.5 μm wide (x–¯ = 24.5 × 2.8 μm, n = 20), 118–143.5 μm long (x–¯ = 129 μm, n = 20), tightly coiled 1.5–2 times, becoming loosely coiled in water, aseptate, guttulate, hyaline, smooth-walled.

#### Culture characteristics.

Conidia germinate on PDA within 14 hours, producing germ tubes from the conidial body. Colonies on PDA are irregular with a raised surface and undulate margin, reaching 3 cm in diameter after 39 days at room temperature (approximately 25 °C), and are brown to dark brown on both the surface and reverse sides.

#### Material examined.

China • Hainan Province, Wuzhishan City, Shuimanhe tropical rainforest scenic area in Wuzhishan, 18°92'N, 109°63'E, on rotting wood in a freshwater habitat, 15 August 2021, Jian Ma, WZS8.2 (HKAS 128942, holotype), ex-type living cultures GZCC 23-0410; • *Ibid*., WZS8.5 (GZAAS 24-0078, paratype), living culture GZCC 24-0164.

#### Notes.

In our phylogenetic tree (Fig. 1), our isolates (GZCC 23-0410 and GZCC 24-0164) formed a sister clade to *N. guizhouensis* (GZCC 23-0725) with 92% ML bootstrap support. *Neohelicomyces wuzhishanensis* (HKAS 128942) can be distinguished from *N. guizhouensis* (KAS 134924) by its wider conidial diameters (23–26 μm *vs.* 18–21.5 μm) (Ma et al. 2024a). Moreover, base pair comparison of *N. wuzhishanensis* (GZCC 23-0410) and *N. guizhouensis* (GZCC 23-0725) shows 31/539 bp differences in ITS (5.8%, gaps 13 bp), 4/530 bp differences in LSU (0.8%, gaps 3 bp), 13/877 bp differences in *tef*1-α (1.5%), and 23/939 bp differences in *rpb*2 (2.4%). Therefore, based on the multigene phylogenetic analysis and morphological differences, we introduce *N. wuzhishanensis* as a novel species.

## ﻿Discussion

*Neohelicomyces* currently comprises 30 species, including the two newly described species, *N. aquisubtropicus* and *N. wuzhishanensis* (Hsieh et al. 2021; Yang et al. 2023; Ma et al. 2024a, b; Peng et al. 2025; Sun et al. 2025).

According to previously published studies, six genera—*Helicodendron*, *Helicoma*, *Helicosporium*, *Neohelicomyces*, *Neohelicosporium*, and *Tubeufia*—represent the most species-rich genera among helicosporous hyphomycetes (Lu and Kang 2020, 2022; Ma et al. 2024a, b; Lu et al. 2025; Peng et al. 2025). The asexual morph of most *Neohelicomyces* species closely resembles that of *Helicomyces*, *Pseudotubeufia*, and *Tubeufia* in having mononematous, septate, pale brown conidiophores; mono- to polyblastic conidiogenous cells; and acropleurogenous or pleurogenous, aseptate or septate, hyaline, helicoid conidia (Yang et al. 2023; Ma et al. 2023, 2024a, b; Peng et al. 2025; Sun et al. 2025). For these morphologically similar helicosporous genera, accurate identification requires a combination of multigene phylogenetic analyses and detailed morphological examination (Linder 1929; Goos 1985, 1986, 1989; Tsui et al. 2006; Zhao et al. 2007; Ruibal et al. 2009; Hyde et al. 2016; Luo et al. 2017; Lu et al. 2018, 2022; Tibpromma et al. 2018; Crous et al. 2019a, b; Dong et al. 2020; Boonmee et al. 2011, 2014, 2021; Hsieh et al. 2021; Yang et al. 2023; Ma et al. 2024a, b; Peng et al. 2025). It is important to note that comparisons of conidial diameter and number of coils among helicosporous species should be based on tightly coiled conidia to ensure consistency and accuracy (Lu et al. 2022, 2023a, b; Xiao et al. 2023; Yang et al. 2023; Ma et al. 2024a, b; Peng et al. 2025).

Some *Neohelicomyces* species exhibit morphological variations across different habitats and geographical regions (Linder 1929; Goos 1989; Tsui et al. 2001; Zhao et al. 2007; Lu et al. 2018; Yang et al. 2023; Ma et al. 2024b). For example, two collections—HMAS 98776 from a terrestrial habitat in Hebei Province and GZAAS 20-0339 from a freshwater habitat in Guizhou Province, China—both identified as *Neohelicomyces pallidus*, show distinct morphological characters (Zhao et al. 2007; Yang et al. 2023). HMAS 98776 differs from GZAAS 20-0339 by possessing smaller conidia (10–16 µm vs. 16–22 µm) and exclusively intercalary conidiogenous cells, whereas GZAAS 20-0339 exhibits both terminal and intercalary conidiogenous cells (Zhao et al. 2007; Yang et al. 2023). These morphological differences are speculated to result from environmental variation across habitats and geographic locations. Therefore, species identification within this genus primarily relies on molecular data, which serve as the principal criterion in taxonomic decision-making. This study enriches our understanding of fungal diversity in subtropical and tropical ecosystems and provides cultures of fungal strains for subsequent research on the secondary metabolites of *Neohelicomyces*.

## Supplementary Material

XML Treatment for Neohelicomyces aquisubtropicus

XML Treatment for Neohelicomyces wuzhishanensis

## ﻿Additional information

### Use of AI

No use of AI was reported.

### Author ORCIDs

Xiao-Yan Ma https://orcid.org/0000-0001-5874-9979

Dan-Dan Song https://orcid.org/0009-0005-3175-6438

Jian Ma https://orcid.org/0009-0008-1291-640X

### Data availability

All of the data that support the findings of this study are available in the main text.

## References

[B1] Boonmee S Zhang Y Chomnunti P Chukeatirote E Tsui CKM Bahkali AH Hyde KD (2011) Revision of lignicolous Tubeufiaceae based on morphological reexamination and phylogenetic analysis. Fungal Diversity 51: 63–102. 10.1007/s13225-011-0147-4

[B2] Boonmee S Rossman AY Liu JK Li WJ Dai DQ Bhat JD Jones EBG McKenzie EHC Xu JC Hyde KD (2014) Tubeufiales, ord. nov., integrating sexual and asexual generic names. Fungal Diversity 68: 239–298. 10.1007/s13225-014-0304-7

[B3] Boonmee S Wanasinghe DN Calabon MS Huanraluek N Chandrasiri SKU Jones GEB Rossi W Leonardi M Singh SK Rana S Singh PN Maurya DK Lagashetti AC Choudhary D Dai YC Zhao CL Mu YH Yuan HS He SH Phookamsak R Jiang HB Martin MP Duenas M Telleria MT Kalucka IL Jagodzinski AM Liimatainen K Pereira DS Phillips AJL Suwannarach N Kumla J Khuna S Lumyong S Potter TB Shivas RG Sparks AH Vaghefi N Abdel-Wahab MA Abdel-Aziz FA Li GJ Lin WF Singh U Bhatt RP Lee HB Nguyen TTT Kirk PM Dutta AK Acharya K Sarma VV Niranjan M Rajeshkumar KC Ashtekar N Lad S Wijayawardene NN Bhat DJ Xu RJ Wijesinghe SN Shen HW Luo ZL Zhang JY Sysouphanthong P Thongklang N Bao DF Aluthmuhandiram JVS Abdollahzadeh J Javadi A Dovana F Usman M Khalid AN Dissanayake AJ Telagathoti A Probst M Peintner U Garrido-Benavent I Bona L Merenyi Z Boros L Zoltan B Stielow JB Jiang N Tian CM Shams E Dehghanizadeh F Pordel A Javan-Nikkhah M Denchev TT Denchev CM Kemler M Begerow D Deng CY Harrower E Bozorov T Kholmuradova T Gafforov Y Abdurazakov A Xu JC Mortimer PE Ren GC Jeewon R Maharachchikumbura SSN Phukhamsakda C Mapook A Hyde KD (2021) Fungal diversity notes 1387–1511: Taxonomic and phylogenetic contributions on genera and species of fungal taxa. Fungal Diversity 111: 1–335. 10.1007/s13225-021-00489-3 34899100 PMC8648402

[B4] Capella-Gutiérrez S Silla-Martínez JM Gabaldón T (2009) trimAl: A tool for automated alignment trimming in large-scale phylogenetic analyses. Bioinformatics 25(15): 1972–1973. 10.1093/bioinformatics/btp348 19505945 PMC2712344

[B5] Chethana KT Manawasinghe IS Hurdeal V Bhunjun CS Appadoo M Gentekaki E Raspé O Promputtha I Hyde KD (2021) What are fungal species and how to delineate them? Fungal Diversity 109: 1–25. 10.1007/s13225-021-00483-9

[B6] Crous P Schumacher RK Akulov A Thangavel R Hernández-Restrepo M Carnegie A Cheewangkoon R Wingfield MJ Summerell BA Quaedvlieg W (2019a) New and interesting fungi. 2. Fungal Systematics and Evolution 3: 57–134. 10.3114/fuse.2019.03.06 32467898 PMC7235984

[B7] Crous PW Wingfield M Lombard L Roets F Swart W Alvarado P Carnegie A Moreno G Luangsaard J Thangavel R (2019b) Fungal Planet description sheets: 951–1041. Persoonia. Molecular Phylogeny and Evolution of Fungi 43: 223–425. 10.3767/persoonia.2019.43.06 PMC7085856 32214501

[B8] Dong W Wang B Hyde KD McKenzie EHC Raja HA Tanaka K Abdel-Wahab MA Abdel-Aziz FA Doilom M Phookamsak R Hongsanan S Wanasinghe DN Yu XD Wang GN Yang H Yang J Thambugala KM Tian Q Luo ZL Yang JB Miller AN Fournier J Boonmee S Hu DM Nalumpang S Zhang H (2020) Freshwater Dothideomycetes. Fungal Diversity 105: 319–575. 10.1007/s13225-020-00463-5

[B9] Goos R (1985) A review of the anamorph genus *Helicomyces*. Mycologia 77: 606–618. 10.1080/00275514.1985.12025146

[B10] Goos R (1986) A review of the anamorph genus *Helicoma*. Mycologia 78: 744–761. 10.1080/00275514.1986.12025318

[B11] Goos R (1989) On the anamorph genera *Helicosporium* and *Drepanospora*. Mycologia 81: 356–374. 10.1080/00275514.1989.12025759

[B12] Hall TA (1999) BioEdit: A user-friendly biological sequence alignment editor and analysis program for Windows 95/98/NT. Nucleic Acids Symposium Series 41: 95–98.

[B13] Hsieh SY Goh TK Kuo CH (2021) New species and records of *Helicosporium* sensu lato from Taiwan, with a reflection on current generic circumscription. Mycological Progress 20: 169–190. 10.1007/s11557-020-01663-8

[B14] Hyde KD Hongsanan S Jeewon R Bhat DJ McKenzie EHC Jones EBG Phookamsak R Ariyawansa HA Boonmee S Zhao Q Abdel-Aziz FA Abdel-Wahab MA Banmai S Chomnunti P Cui BK Daranagama DA Das K Dayarathne MC de Silva NI Dissanayake AJ Doilom M Ehanayaka AH Gibertoni TB Go’es-Neto A Huang SK Jayasiri SC Jayawardena RS Konta S Lee HB Li WJ Lin CG Liu JK Lu YZ Luo ZL Manawasinghe IS Manimohan P Mapook A Niskanen T Norphanphoun C Papizadeh M Perera RH Phukhamsakda C Richter C de Santiago ALCM Drechsler-Santos ER Senanayake IC Tanaka K Tennakoon TMDS Thambugala KM Tian Q Tibpromma S Thongbai B Vizzini A Wanasinghe DN Wijayawardene NN Wu HX Yang J Zeng XY Zhang H Zhang JF Bulgakov TS Camporesi E Bahkali AH Amoozegar MA Araujo-Neta LS Ammirati JF Baghela A Bhatt RP Bojantchev D Buyck B de Silva GA de Lima CLF de Oliveira RJV de Souza CAF Dai YC Dima B Duong TT Ercole E Mafalda-Freire F Ghosh A Hashimoto A Kamolhan S Kang JC Karunarathna SC Kirk PM Kyto¨vuori I Lantieri A Liimatainen K Liu ZY Liu XZ Lu¨cking R Medardi G Mortimer PE Nguyen TTT Promputtha I Raj KNA Reck MA Lumyong S Shahzadeh-Fazeli SA Stadler M Soudi MR Su HY Takahashi T Tangthirasunun N Uniyal P Wang Y Wen TC Xu JC Zhang ZK Zhao YC Zhou JL Zhu L (2016) Fungal diversity notes 367–490: Taxonomic and phylogenetic contributions to fungal taxa. Fungal Diversity 80: 1–270. 10.1007/s13225-016-0373-x

[B15] Katoh K Rozewicki J Yamada KD (2019) MAFFT online service: Multiple sequence alignment, interactive sequence choice and visualization. Briefings in Bioinformatics 20: 1160–1166. 10.1093/bib/bbx108 28968734 PMC6781576

[B16] Larsson A (2014) AliView: A fast and lightweight alignment viewer and editor for large datasets. Bioinformatics 30: 3276–3278. 10.1093/bioinformatics/btu531 25095880 PMC4221126

[B17] Linder DH (1929) A monograph of the helicosporous fungi imperfecti. Annals of the Missouri Botanical Garden 16: 227–388. 10.2307/2394038

[B18] Liu YJ Whelen S Hall BD (1999) Phylogenetic relationships among ascomycetes: Evidence from an RNA polymerse II subunit. Molecular Biology and Evolution 16(12): 1799–1808. 10.1093/oxfordjournals.molbev.a026092 10605121

[B19] Lu YZ Kang JC (2020) Research progress on helicosporous hyphomycetes. Journal of Fungal Research 18(4): 304–314. 10.13341/j.jfr.2020.8012

[B20] Lu YZ Liu JK Hyde KD Jeewon R Kang JC Fan C Boonmee S Bhat DJ Luo ZL Lin CG (2018) A taxonomic reassessment of Tubeufiales based on multi-locus phylogeny and morphology. Fungal Diversity 92: 131–344. 10.1007/s13225-018-0411-y

[B21] Lu YZ Ma J Xiao XJ Zhang LJ Xiao YP Kang JC (2022) Four new species and three new records of helicosporous hyphomycetes from China and their multi-gene phylogenies. Frontiers in Microbiology 13: e1053849. 10.3389/fmicb.2022.1053849 PMC9732463 36504835

[B22] Lu YZ Ma J Xiao XJ Zhang LJ Ma XY Xiao YP Kang JC (2023a) Two novel species and one new record of *Helicoma* from tropical China. Junwu Xuebao 42(1): 263–277. 10.13346/j.mycosystema.220445

[B23] Lu YZ Ma J Xiao XJ Zhang LJ Xiao YP Kang JC (2023b) Morphology and phylogeny of *Tubeufia liyui* sp. nov. Journal of Fungal Research 21(1/2/3): 14–23. 10.13341/j.jfr.2023.1582

[B24] Lu L Karunarathna SC Xiong YR Han LS Chen XM Xu RF Liu XF Zeng XY Dai DQ Elgorban AM Jayawardena RS Hyde KD Tibpromma S (2025) Taxonomy and systematics of micro-fungi associated with *Coffea* in southern China and northern Thailand. Fungal Diversity proof. 10.21203/rs.3.rs-6462360/v1

[B25] Luo ZL Bhat DJ Jeewon R Boonmee S Bao DF Zhao YC Chai HM Su HY Su XJ Hyde KD (2017) Molecular phylogeny and morphological characterization of asexual fungi (Tubeufiaceae) from freshwater habitats in Yunnan, China. Cryptogamie. Mycologie 38(1): 27–53. 10.7872/crym/v38.iss1.2017.27

[B26] Ma J Xiao XJ Liu NG Boonmee S Xiao YP Lu YZ (2023) Morphological and multi-gene phylogenetic analyses reveal *Pseudotubeufia* gen. nov. and two new species in Tubeufiaceae from China. Journal of Fungi 9: e742. 10.3390/jof9070742 PMC10381972 37504731

[B27] Ma J Gomdola D Boonmee S Shen HW Tang X Zhang LJ Lu YZ Hyde KD (2024a) Three new species of *Neohelicomyces* (Tubeufiales, Tubeufiaceae) from freshwater and terrestrial habitats in China. MycoKeys 105: 317–336. 10.3897/mycokeys.105.124129 38863446 PMC11165267

[B28] Ma J Hyde KD Tibpromma S Gomdola D Liu NG Norphanphoun C Bao DF Boonmee S Xiao XJ Zhang LJ Luo ZL Zhao Q Suwannarach N Karunarathna SC Liu JK Lu YZ (2024b) Taxonomy and systematics of lignicolous helicosporous hyphomycetes. Fungal Diversity 129: 365–653. 10.1007/s13225-024-00544-9

[B29] Nylander JAA Zoology S Posada D Mrmodeltest R Os F (2008) MrModeltest2 v. 2.3 (Program for Selecting DNA Substitution Models Using PAUP*). Evolutionary Biology Centre, Uppsala, Sweden.

[B30] Nguyen LT Schmidt HA Von Haeseler A Minh BQ (2015) IQ-TREE: a fast and effective stochastic algorithm for estimating maximum-likelihood phylogenies. Molecular Biology and Evolution 32(1): 268–274. 10.1093/molbev/msu300 25371430 PMC4271533

[B31] Peng T Lu YZ Bai S Zhang JY Xiao XJ Wu N Ma J (2025) Novel *Helicosporium* and *Neohelicomyces* (Tubeufiaceae, Tubeufiales) species from terrestrial habitats in China and Thailand. MycoKeys 112: 81–101. 10.3897/mycokeys.112.140211 39830363 PMC11742100

[B32] Rathnayaka AR Tennakoon DS Jones GE Wanasinghe DN Bhat DJ Priyashantha AH Stephenson SL Tibpromma S Karunarathna SC (2024) Significance of precise documentation of hosts and geospatial data of fungal collections, with an emphasis on plant-associated fungi. New Zealand Journal of Botany 63(2–3): 462–489. 10.1080/0028825X.2024.2381734

[B33] Rehner SA Buckley E (2005) A beauveria phylogeny inferred from nuclear ITS and EF1-α sequences: Evidence for cryptic diversification and links to *Cordyceps* teleomorphs. Mycologia 97: 84–98. 10.3852/mycologia.97.1.84 16389960

[B34] Ruibal C Gueidan C Selbmann L Gorbushina AA Crous PW Groenewald J Muggia L Grube M Isola D Schoch CL (2009) Phylogeny of rock-inhabiting fungi related to Dothideomycetes. Studies in Mycology 64: 123–133. 10.3114/sim.2009.64.06 20169026 PMC2816969

[B35] Senanayake IC Rathnayaka AR Marasinghe DS Calabon MS Gentekaki E Lee HB Hurdeal VG Pem D Dissanayake LS Wijesinghe SN Bundhun D Nguyen TT Goonasekara ID Abeywickrama PD Bhunjun CS Jayawardena RS Wanasinghe DN Jeewon R Bhat DJ Xiang MM (2020) Morphological approaches in studying fungi: Collection, examination, isolation, sporulation and preservation. Mycosphere : Journal of Fungal Biology 11(1): 2678–2754. 10.5943/mycosphere/11/1/20

[B36] Sun YR Hyde KD Liu NG Jayawardena RS Wijayawardene NN Ma J Zhang Q Al-Otibi F Wang Y (2025) Micro-fungi in southern China and northern Thailand: Emphasis on medicinal plants. Fungal Diversity 131: 99–299. 10.1007/s13225-024-00549-4

[B37] Swindell SR Plasterer TN (1997) Seqman. Sequence Data Analysis Guidebook. Springer, 75–89. 10.1385/0-89603-358-9:75

[B38] Tibpromma S Hyde KD McKenzie EHC Bhat DJ Phillips AJL Wanasinghe DN Samarakoon MC Jayawardena RS Dissanayake AJ Tennakoon DS Doilom M Phookamsak R Tang AMC Xu JC Mortimer PE Promputtha I Maharachchikumbura SSN Khan S Karunarathna SC (2018) Fungal diversity notes 840–928: Micro-fungi associated with Pandanaceae. Fungal Diversity 93: 1–160. 10.1007/s13225-018-0408-6

[B39] Tsui CK Hyde KD Hodgkiss IJ (2001) Longitudinal and temporal distribution of freshwater ascomycetes and dematiaceous hyphomycetes on submerged wood in the Lam Tsuen River, Hong Kong. Journal of the North American Benthological Society 20(4): 533–549. 10.2307/1468086

[B40] Tsui CK Sivichai S Berbee ML (2006) Molecular systematics of *Helicoma*, *Helicomyces* and *Helicosporium* and their teleomorphs inferred from rDNA sequences. Mycologia 98: 94–104. 10.3852/mycologia.98.1.94 16800307

[B41] Vaidya G Lohman DJ Meier R (2011) SequenceMatrix: Concatenation software for the fast assembly of multi-gene datasets with character set and codon information. Cladistics 27: 171–180. 10.1111/j.1096-0031.2010.00329.x 34875773

[B42] Vilgalys R Hester M (1990) Rapid genetic identification and mapping of enzymatically amplified ribosomal DNA from several *Cryptococcus* species. Journal of Bacteriology 172(8): 4238–4246. 10.1128/jb.172.8.4238-4246.1990 2376561 PMC213247

[B43] White TJ Bruns T Lee S Taylor J (1990) Amplification and direct sequencing of fungal ribosomal RNA genes for phylogenetics. PCR Protocols: A Guide to Methods and Applications, 315–322. 10.1016/B978-0-12-372180-8.50042-1

[B44] Xiao XJ Ma J Zhang LJ Liu NG Xiao YP Tian XG Luo ZL Lu YZ (2023) Additions to the genus *Helicosporium* (Tubeufiaceae, Tubeufiales) from China with an identification key to *Helicosporium* taxa. Journal of Fungi 9: e775. 10.3390/jof9070775 PMC10381633 37504763

[B45] Yang J Liu LL Jones EBG Hyde KD Liu ZY Bao DF Liu NG Li WL Shen HW Yu XD (2023) Freshwater fungi from karst landscapes in China and Thailand. Fungal Diversity 119: 1–212. 10.1007/s13225-023-00514-7

[B46] Zhao GZ Liu XZ Wu WP (2007) Helicosporous hyphomycetes from China. Fungal Diversity 26: 313–524.

[B47] Zheng W Han L He ZJ Kang JC (2023) A new alkaloid derivative from the saprophytic fungus *Neohelicomyces hyalosporus* PF11-1. Natural Product Research 1–5. 10.1080/14786419.2023.2167202 36644981

